# Poly[tetra­aqua­bis­(μ_3_-oxalato-κ^5^ *O* ^1^,*O* ^2^:*O* ^1′^:*O* ^1′^,*O* ^2′^)(μ_2_-oxalato-κ^4^ *O* ^1^,*O* ^2^:*O* ^1′^,*O* ^2′^)dipraseodymium(III)]

**DOI:** 10.1107/S1600536812011014

**Published:** 2012-03-17

**Authors:** Cheng-Jun Hao, Hui Xie

**Affiliations:** a College of Chemistry and Chemical Engineering, Pingdingshan University, Pingdingshan 467000, People’s Republic of China

## Abstract

In the title complex, [Pr_2_(C_2_O_4_)_3_(H_2_O)_4_]_*n*_, the two independent Pr^III^ ions are both nine-coordinated in a distorted monocapped square-anti­prismatic geometry by seven O atoms from four oxalate ligands and two water mol­ecules. The Pr^III^ ions are bridged by the oxalate ligands, forming a layer parallel to (001). O—H⋯O hydrogen bonds connect the layers.

## Related literature

For the structures and potential applications of lanthanide complexes, see: Ma *et al.* (2001 ▶); Shibasaki & Yoshikawa (2002 ▶); Song *et al.* (2012 ▶).
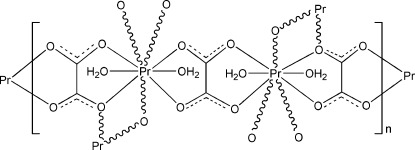


## Experimental

### 

#### Crystal data

[Pr_2_(C_2_O_4_)_3_(H_2_O)_4_]
*M* *_r_* = 617.94
Orthorhombic, 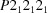

*a* = 8.6358 (17) Å
*b* = 9.5356 (19) Å
*c* = 16.885 (3) Å
*V* = 1390.4 (5) Å^3^
*Z* = 4
Mo *K*α radiation
μ = 7.02 mm^−1^
*T* = 293 K
0.23 × 0.22 × 0.20 mm

#### Data collection

Rigaku Mercury CCD diffractometer
Absorption correction: multi-scan (*CrystalClear*; Rigaku, 2002 ▶) *T*_min_ = 0.295, *T*_max_ = 0.334
13654 measured reflections
3181 independent reflections
2826 reflections with *I* > 2σ(*I*)
*R*_int_ = 0.047

#### Refinement

*R*[*F* ^2^ > 2σ(*F* ^2^)] = 0.029
*wR*(*F* ^2^) = 0.072
*S* = 1.04
3181 reflections
217 parameters
H-atom parameters constrained
Δρ_max_ = 1.34 e Å^−3^
Δρ_min_ = −1.44 e Å^−3^
Absolute structure: Flack (1983 ▶), 1344 Friedel pairs
Flack parameter: 0.49 (3)

### 

Data collection: *CrystalClear* (Rigaku, 2002 ▶); cell refinement: *CrystalClear*; data reduction: *CrystalStructure* (Rigaku/MSC, 2002 ▶); program(s) used to solve structure: *SHELXS97* (Sheldrick, 2008 ▶); program(s) used to refine structure: *SHELXL97* (Sheldrick, 2008 ▶); molecular graphics: *ORTEPII* (Johnson, 1976 ▶); software used to prepare material for publication: *SHELXL97*.

## Supplementary Material

Crystal structure: contains datablock(s) I, global. DOI: 10.1107/S1600536812011014/hy2521sup1.cif

Structure factors: contains datablock(s) I. DOI: 10.1107/S1600536812011014/hy2521Isup2.hkl

Additional supplementary materials:  crystallographic information; 3D view; checkCIF report

## Figures and Tables

**Table 1:** Hydrogen-bond geometry (Å, °)

*D*—H⋯*A*	*D*—H	H⋯*A*	*D*⋯*A*	*D*—H⋯*A*
O1*W*—H1*W*⋯O2^i^	0.85	2.02	2.852 (6)	166
O1*W*—H2*W*⋯O8^ii^	0.85	2.15	2.998 (6)	173
O2*W*—H3*W*⋯O4^iii^	0.85	2.40	2.998 (6)	128
O2*W*—H4*W*⋯O6^iv^	0.85	2.01	2.792 (6)	152
O3*W*—H5*W*⋯O12^v^	0.85	1.97	2.780 (6)	158
O3*W*—H6*W*⋯O3^v^	0.85	2.60	3.379 (7)	154
O4*W*—H7*W*⋯O1^vi^	0.85	2.16	2.865 (6)	140
O4*W*—H8*W*⋯O9^v^	0.85	2.04	2.882 (6)	169

Symmetry codes: (i) 
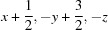
; (ii) 
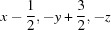
; (iii) 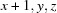; (iv) 
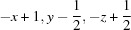
; (v) 
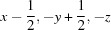
; (vi) 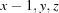.

**Fig. 1.**
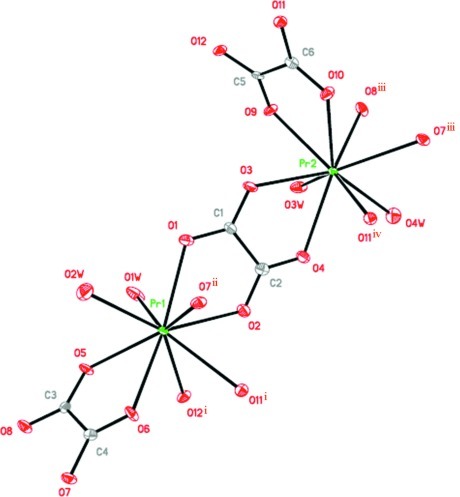
The asymmetric unit of the title compound. Displacement ellipsoids are drawn at the 30% probability level. H atoms are omitted for clarity. [Symmetry codes: (i) x, y+1, z; (ii) -x+1, y-1/2, -z+1/2; (iii) x-1, y-1, z; (iv) -x, y+1/2, -z+1/2.]

## supplementary crystallographic information

### Comment

During the past decade, considerable efforts have been devoted to the design and construction of new lanthanide coordination polymers due to their intriguing structural diversity and potential applications in many areas (Ma *et al.*, 2001; Shibasaki & Yoshikawa, 2002; Song *et al.*, 2012). Oxalate owning four carboxylate O atoms is highly accessible to lanthanide ions to form novel structures.

As shown in Fig. 1, in the asymmetric unit of the title complex, there are two independent Pr^III^ ions with a similar coordination environment. Each Pr^III^ ion is nine-coordinated by seven O atoms from four oxalate ligands and two O atoms from two terminal water molecules. The Pr1 and Pr2 atoms are bridged by two carboxylate O atoms, forming a Pr_2_O_2_ subunit with a Pr···Pr distance of 4.2893 (7) Å. Such subunits are connected by the oxalate ligands, generating a layer parallel to (0 0 1). It is noted that the oxalate ligands exhibit two kinds of coordination modes: one adopts a bisbidentate coordination mode bridging two Pr^III^ ions; the other adopts a chelating and bridging coordination mode connecting three Pr^III^ ions. The adjacent layers are further linked into a three-dimensional network *via* intermolecular O—H···O hydrogen bonds (Table 1).

### Experimental

A mixture of Pr(NO_3_)_3_.6H_2_O (0.5 mmol, 0.217 g) and oxalic acid (1 mmol, 0.09 g) in 10 ml of H_2_O was sealed in an autoclave equipped with a Teflon liner (30 ml) and then heated to 453 K for 4 days. After gradual cooling to room temperature, crystals were obtained and collected by filtration with a yield of 31% based on Pr.

### Refinement

H atoms of water molecules were located in a difference Fourier map and refined as riding, with O—H = 0.85 Å and with *U*_iso_(H) = 1.5*U*_eq_(O). The highest residual electron density was found at 0.82 Å from Pr1 atom and the deepest hole at 0.80 Å from Pr2 atom.

### Crystal data

**Table:**

[Pr_2_(C_2_O_4_)_3_(H_2_O)_4_]	*F*(000) = 1160
*M**_r_* = 617.94	*D*_x_ = 2.952 Mg m^−^^3^
Orthorhombic, *P*2_1_2_1_2_1_	Mo *K*α radiation, λ = 0.71073 Å
Hall symbol: P 2ac 2ab	Cell parameters from 3600 reflections
*a* = 8.6358 (17) Å	θ = 1.4–28.0°
*b* = 9.5356 (19) Å	µ = 7.02 mm^−^^1^
*c* = 16.885 (3) Å	*T* = 293 K
*V* = 1390.4 (5) Å^3^	Block, green
*Z* = 4	0.23 × 0.22 × 0.20 mm

### Data collection

**Table:**

Rigaku Mercury CCD diffractometer	3181 independent reflections
Radiation source: fine-focus sealed tube	2826 reflections with *I* > 2σ(*I*)
Graphite monochromator	*R*_int_ = 0.047
ω scans	θ_max_ = 27.4°, θ_min_ = 3.2°
Absorption correction: multi-scan (*CrystalClear*; Rigaku, 2002)	*h* = −9→11
*T*_min_ = 0.295, *T*_max_ = 0.334	*k* = −12→12
13654 measured reflections	*l* = −21→21

### Refinement

**Table:**

Refinement on *F*^2^	Secondary atom site location: difference Fourier map
Least-squares matrix: full	Hydrogen site location: inferred from neighbouring sites
*R*[*F*^2^ > 2σ(*F*^2^)] = 0.029	H-atom parameters constrained
*wR*(*F*^2^) = 0.072	*w* = 1/[σ^2^(*F*_o_^2^) + (0.0284*P*)^2^ + 3.201*P*] where *P* = (*F*_o_^2^ + 2*F*_c_^2^)/3
*S* = 1.04	(Δ/σ)_max_ = 0.001
3181 reflections	Δρ_max_ = 1.34 e Å^−^^3^
217 parameters	Δρ_min_ = −1.44 e Å^−^^3^
0 restraints	Absolute structure: Flack (1983), 1344 Friedel pairs
Primary atom site location: structure-invariant direct methods	Flack parameter: 0.49 (3)

### Special details

**Table:**

Geometry. All e.s.d.'s (except the e.s.d. in the dihedral angle between two l.s. planes) are estimated using the full covariance matrix. The cell e.s.d.'s are taken into account individually in the estimation of e.s.d.'s in distances, angles and torsion angles; correlations between e.s.d.'s in cell parameters are only used when they are defined by crystal symmetry. An approximate (isotropic) treatment of cell e.s.d.'s is used for estimating e.s.d.'s involving l.s. planes.
Refinement. Refinement of *F*^2^ against ALL reflections. The weighted *R*-factor *wR* and goodness of fit *S* are based on *F*^2^, conventional *R*-factors *R* are based on *F*, with *F* set to zero for negative *F*^2^. The threshold expression of *F*^2^ > σ(*F*^2^) is used only for calculating *R*-factors(gt) *etc*. and is not relevant to the choice of reflections for refinement. *R*-factors based on *F*^2^ are statistically about twice as large as those based on *F*, and *R*- factors based on ALL data will be even larger.

### Fractional atomic coordinates and isotropic or equivalent isotropic displacement parameters (Å^2^)

**Table:**

	*x*	*y*	*z*	*U*_iso_*/*U*_eq_	
Pr1	0.37510 (4)	0.75389 (2)	0.143154 (14)	0.01214 (9)	
Pr2	−0.12589 (4)	0.26126 (3)	0.137142 (14)	0.01185 (8)	
O1	0.3263 (5)	0.4984 (4)	0.1336 (3)	0.0245 (10)	
O2	0.1023 (5)	0.6852 (4)	0.1081 (2)	0.0220 (9)	
O3	0.1479 (5)	0.3304 (5)	0.1473 (3)	0.0383 (12)	
O4	−0.0761 (5)	0.5159 (5)	0.1102 (3)	0.0236 (10)	
O5	0.5734 (5)	0.8913 (4)	0.0744 (2)	0.0220 (9)	
O6	0.4832 (5)	0.9677 (5)	0.2184 (2)	0.0221 (9)	
O7	0.6490 (5)	1.1490 (4)	0.2172 (2)	0.0209 (9)	
O8	0.7467 (5)	1.0643 (5)	0.0716 (3)	0.0255 (10)	
O9	0.0572 (5)	0.0999 (5)	0.0683 (2)	0.0205 (9)	
O10	−0.0198 (5)	0.0560 (4)	0.2191 (2)	0.0230 (9)	
O11	0.1596 (4)	−0.1155 (4)	0.2289 (2)	0.0186 (9)	
O12	0.2521 (5)	−0.0484 (4)	0.0791 (2)	0.0219 (9)	
O1W	0.4097 (7)	0.6778 (5)	0.0052 (2)	0.0361 (14)	
H1W	0.4747	0.7253	−0.0215	0.054*	
H2W	0.3561	0.6140	−0.0168	0.054*	
O2W	0.6418 (6)	0.6597 (5)	0.1743 (3)	0.0357 (11)	
H3W	0.7010	0.6599	0.1341	0.053*	
H4W	0.6369	0.5950	0.2088	0.053*	
O3W	−0.1196 (7)	0.3125 (5)	−0.0081 (2)	0.0296 (10)	
H5W	−0.1447	0.3960	−0.0200	0.044*	
H6W	−0.1567	0.2530	−0.0404	0.044*	
O4W	−0.3828 (6)	0.3679 (5)	0.0986 (2)	0.0303 (10)	
H7W	−0.4443	0.4093	0.1304	0.045*	
H8W	−0.4120	0.3834	0.0513	0.045*	
C1	0.1907 (7)	0.4536 (6)	0.1341 (4)	0.0214 (13)	
C2	0.0597 (7)	0.5610 (6)	0.1160 (3)	0.0196 (12)	
C3	0.6393 (7)	0.9949 (6)	0.1035 (3)	0.0160 (12)	
C4	0.5861 (6)	1.0398 (6)	0.1870 (3)	0.0169 (12)	
C5	0.1355 (7)	0.0127 (5)	0.1057 (3)	0.0164 (11)	
C6	0.0863 (6)	−0.0190 (6)	0.1925 (3)	0.0166 (12)	

### Atomic displacement parameters (Å^2^)

**Table:**

	*U*^11^	*U*^22^	*U*^33^	*U*^12^	*U*^13^	*U*^23^
Pr1	0.01379 (15)	0.00894 (14)	0.01370 (14)	−0.00143 (13)	0.00092 (11)	0.00098 (9)
Pr2	0.01324 (15)	0.00861 (13)	0.01371 (14)	−0.00137 (13)	0.00076 (12)	−0.00112 (9)
O1	0.025 (2)	0.013 (2)	0.035 (2)	−0.0023 (16)	−0.001 (2)	0.0004 (18)
O2	0.019 (2)	0.019 (2)	0.028 (2)	−0.0007 (18)	−0.0038 (19)	0.0043 (16)
O3	0.018 (2)	0.0103 (19)	0.086 (4)	−0.0033 (18)	−0.005 (2)	0.009 (2)
O4	0.022 (2)	0.018 (2)	0.031 (2)	−0.0029 (16)	−0.0023 (19)	0.0017 (18)
O5	0.030 (2)	0.015 (2)	0.020 (2)	−0.0103 (17)	0.0063 (18)	−0.0088 (17)
O6	0.025 (2)	0.025 (2)	0.0165 (18)	−0.0139 (18)	0.0081 (18)	−0.0051 (18)
O7	0.028 (2)	0.0162 (19)	0.0188 (18)	−0.0058 (17)	0.0033 (19)	−0.0052 (15)
O8	0.030 (2)	0.022 (3)	0.024 (2)	−0.0126 (19)	0.010 (2)	−0.0098 (19)
O9	0.025 (2)	0.020 (2)	0.0162 (19)	0.0110 (18)	0.0016 (18)	0.0036 (17)
O10	0.027 (2)	0.024 (2)	0.019 (2)	0.0093 (18)	0.0056 (19)	0.0051 (19)
O11	0.021 (2)	0.019 (2)	0.0155 (18)	0.0023 (16)	−0.0005 (17)	0.0045 (15)
O12	0.027 (2)	0.017 (2)	0.022 (2)	0.0088 (18)	0.0093 (18)	0.0092 (18)
O1W	0.056 (4)	0.027 (3)	0.025 (2)	−0.023 (2)	0.016 (2)	−0.0094 (17)
O2W	0.030 (3)	0.030 (3)	0.046 (3)	0.008 (2)	0.003 (3)	0.006 (2)
O3W	0.046 (3)	0.020 (2)	0.024 (2)	0.007 (3)	0.002 (2)	0.0062 (15)
O4W	0.027 (2)	0.038 (3)	0.027 (2)	0.008 (2)	−0.001 (2)	0.0004 (17)
C1	0.022 (3)	0.013 (3)	0.029 (3)	−0.003 (2)	0.002 (3)	0.000 (3)
C2	0.027 (3)	0.018 (3)	0.014 (3)	−0.001 (2)	0.000 (2)	−0.001 (2)
C3	0.016 (3)	0.015 (3)	0.017 (3)	0.002 (3)	0.004 (3)	−0.0010 (18)
C4	0.021 (3)	0.014 (3)	0.015 (3)	−0.001 (2)	−0.001 (2)	−0.002 (2)
C5	0.022 (3)	0.009 (3)	0.018 (3)	0.000 (3)	0.000 (3)	0.0023 (18)
C6	0.020 (3)	0.016 (3)	0.014 (2)	−0.001 (2)	−0.002 (2)	0.002 (2)

### Geometric parameters (Å, º)

**Table:**

Pr1—O12^i^	2.419 (4)	O4—C2	1.253 (7)
Pr1—O5	2.449 (4)	O5—C3	1.241 (6)
Pr1—O1W	2.458 (4)	O6—C4	1.242 (7)
Pr1—O1	2.478 (4)	O7—C4	1.281 (7)
Pr1—O2	2.516 (4)	O8—C3	1.261 (7)
Pr1—O2W	2.527 (5)	O9—C5	1.244 (7)
Pr1—O7^ii^	2.570 (4)	O10—C6	1.247 (7)
Pr1—O6	2.578 (4)	O11—C6	1.275 (7)
Pr1—O11^i^	2.666 (4)	O12—C5	1.247 (7)
Pr2—O8^iii^	2.442 (4)	O1W—H1W	0.8500
Pr2—O3	2.460 (4)	O1W—H2W	0.8501
Pr2—O9	2.494 (4)	O2W—H3W	0.8500
Pr2—O3W	2.501 (4)	O2W—H4W	0.8500
Pr2—O4	2.508 (4)	O3W—H5W	0.8500
Pr2—O4W	2.526 (5)	O3W—H6W	0.8499
Pr2—O11^iv^	2.565 (4)	O4W—H7W	0.8511
Pr2—O10	2.566 (4)	O4W—H8W	0.8513
Pr2—O7^iii^	2.598 (4)	C1—C2	1.556 (9)
O1—C1	1.247 (7)	C3—C4	1.544 (7)
O2—C2	1.247 (7)	C5—C6	1.555 (8)
O3—C1	1.252 (7)		
			
O12^i^—Pr1—O5	71.23 (15)	O9—Pr2—O10	63.55 (13)
O12^i^—Pr1—O1W	81.93 (16)	O3W—Pr2—O10	132.08 (14)
O5—Pr1—O1W	67.89 (15)	O4—Pr2—O10	140.82 (14)
O12^i^—Pr1—O1	131.49 (15)	O4W—Pr2—O10	139.42 (14)
O5—Pr1—O1	127.89 (14)	O11^iv^—Pr2—O10	85.07 (13)
O1W—Pr1—O1	70.66 (15)	O8^iii^—Pr2—O7^iii^	65.29 (13)
O12^i^—Pr1—O2	71.72 (14)	O3—Pr2—O7^iii^	142.48 (15)
O5—Pr1—O2	133.05 (13)	O9—Pr2—O7^iii^	117.55 (13)
O1W—Pr1—O2	79.30 (16)	O3W—Pr2—O7^iii^	127.34 (15)
O1—Pr1—O2	64.51 (14)	O4—Pr2—O7^iii^	128.43 (13)
O12^i^—Pr1—O2W	140.37 (15)	O4W—Pr2—O7^iii^	69.05 (14)
O5—Pr1—O2W	69.61 (15)	O11^iv^—Pr2—O7^iii^	69.21 (11)
O1W—Pr1—O2W	88.94 (17)	O10—Pr2—O7^iii^	70.88 (13)
O1—Pr1—O2W	79.57 (15)	C1—O1—Pr1	119.7 (4)
O2—Pr1—O2W	144.08 (15)	C2—O2—Pr1	119.9 (4)
O12^i^—Pr1—O7^ii^	132.68 (13)	C1—O3—Pr2	121.5 (4)
O5—Pr1—O7^ii^	134.42 (14)	C2—O4—Pr2	118.6 (4)
O1W—Pr1—O7^ii^	139.87 (15)	C3—O5—Pr1	123.9 (4)
O1—Pr1—O7^ii^	70.32 (14)	C4—O6—Pr1	119.1 (3)
O2—Pr1—O7^ii^	92.21 (13)	C4—O7—Pr1^v^	130.3 (3)
O2W—Pr1—O7^ii^	75.20 (14)	C4—O7—Pr2^vi^	116.5 (3)
O12^i^—Pr1—O6	76.29 (15)	Pr1^v^—O7—Pr2^vi^	112.19 (14)
O5—Pr1—O6	63.73 (12)	C3—O8—Pr2^vi^	122.8 (3)
O1W—Pr1—O6	131.05 (14)	C5—O9—Pr2	121.3 (4)
O1—Pr1—O6	150.63 (14)	C6—O10—Pr2	120.4 (3)
O2—Pr1—O6	131.37 (13)	C6—O11—Pr2^vii^	134.4 (3)
O2W—Pr1—O6	81.29 (14)	C6—O11—Pr1^viii^	114.9 (3)
O7^ii^—Pr1—O6	83.38 (13)	Pr2^vii^—O11—Pr1^viii^	110.13 (14)
O12^i^—Pr1—O11^i^	64.69 (12)	C5—O12—Pr1^viii^	123.9 (3)
O5—Pr1—O11^i^	119.72 (13)	Pr1—O1W—H1W	115.2
O1W—Pr1—O11^i^	137.50 (16)	Pr1—O1W—H2W	124.0
O1—Pr1—O11^i^	112.07 (13)	H1W—O1W—H2W	120.6
O2—Pr1—O11^i^	66.14 (12)	Pr1—O2W—H3W	112.4
O2W—Pr1—O11^i^	133.55 (12)	Pr1—O2W—H4W	110.8
O7^ii^—Pr1—O11^i^	68.09 (12)	H3W—O2W—H4W	125.4
O6—Pr1—O11^i^	67.40 (12)	Pr2—O3W—H5W	114.3
O8^iii^—Pr2—O3	132.12 (15)	Pr2—O3W—H6W	119.4
O8^iii^—Pr2—O9	66.40 (15)	H5W—O3W—H6W	112.1
O3—Pr2—O9	65.72 (15)	Pr2—O4W—H7W	124.9
O8^iii^—Pr2—O3W	73.46 (15)	Pr2—O4W—H8W	124.9
O3—Pr2—O3W	89.72 (18)	H7W—O4W—H8W	109.1
O9—Pr2—O3W	69.50 (14)	O1—C1—O3	126.9 (6)
O8^iii^—Pr2—O4	137.69 (15)	O1—C1—C2	117.2 (5)
O3—Pr2—O4	65.69 (14)	O3—C1—C2	116.0 (5)
O9—Pr2—O4	113.85 (14)	O2—C2—O4	126.4 (6)
O3W—Pr2—O4	68.22 (15)	O2—C2—C1	115.6 (5)
O8^iii^—Pr2—O4W	78.30 (16)	O4—C2—C1	118.0 (5)
O3—Pr2—O4W	138.94 (15)	O5—C3—O8	125.9 (5)
O9—Pr2—O4W	133.26 (13)	O5—C3—C4	116.5 (5)
O3W—Pr2—O4W	71.82 (15)	O8—C3—C4	117.6 (5)
O4—Pr2—O4W	73.38 (14)	O6—C4—O7	125.7 (5)
O8^iii^—Pr2—O11^iv^	134.49 (13)	O6—C4—C3	116.7 (5)
O3—Pr2—O11^iv^	85.69 (15)	O7—C4—C3	117.6 (5)
O9—Pr2—O11^iv^	139.96 (13)	O9—C5—O12	124.6 (5)
O3W—Pr2—O11^iv^	141.03 (13)	O9—C5—C6	117.4 (5)
O4—Pr2—O11^iv^	74.70 (14)	O12—C5—C6	118.0 (5)
O4W—Pr2—O11^iv^	86.72 (13)	O10—C6—O11	127.2 (5)
O8^iii^—Pr2—O10	79.57 (15)	O10—C6—C5	115.4 (5)
O3—Pr2—O10	79.85 (14)	O11—C6—C5	117.3 (5)
			
O12^i^—Pr1—O1—C1	−43.6 (5)	O4W—Pr2—O9—C5	−146.9 (4)
O5—Pr1—O1—C1	−141.8 (4)	O11^iv^—Pr2—O9—C5	29.4 (5)
O1W—Pr1—O1—C1	−103.0 (5)	O10—Pr2—O9—C5	−12.7 (4)
O2—Pr1—O1—C1	−15.9 (4)	O7^iii^—Pr2—O9—C5	−60.5 (5)
O2W—Pr1—O1—C1	164.5 (5)	O8^iii^—Pr2—O10—C6	77.6 (4)
O7^ii^—Pr1—O1—C1	86.6 (5)	O3—Pr2—O10—C6	−58.9 (4)
O6—Pr1—O1—C1	114.3 (5)	O9—Pr2—O10—C6	8.9 (4)
O11^i^—Pr1—O1—C1	31.5 (5)	O3W—Pr2—O10—C6	21.4 (5)
O12^i^—Pr1—O2—C2	171.5 (4)	O4—Pr2—O10—C6	−87.1 (5)
O5—Pr1—O2—C2	132.1 (4)	O4W—Pr2—O10—C6	135.4 (4)
O1W—Pr1—O2—C2	86.6 (4)	O11^iv^—Pr2—O10—C6	−145.4 (4)
O1—Pr1—O2—C2	13.0 (4)	O7^iii^—Pr2—O10—C6	144.9 (5)
O2W—Pr1—O2—C2	13.6 (5)	Pr1—O1—C1—O3	−163.0 (6)
O7^ii^—Pr1—O2—C2	−53.9 (4)	Pr1—O1—C1—C2	17.5 (7)
O6—Pr1—O2—C2	−137.1 (4)	Pr2—O3—C1—O1	−176.1 (5)
O11^i^—Pr1—O2—C2	−118.8 (4)	Pr2—O3—C1—C2	3.5 (8)
O8^iii^—Pr2—O3—C1	132.1 (5)	Pr1—O2—C2—O4	170.7 (5)
O9—Pr2—O3—C1	132.4 (6)	Pr1—O2—C2—C1	−9.8 (7)
O3W—Pr2—O3—C1	64.9 (5)	Pr2—O4—C2—O2	−176.5 (5)
O4—Pr2—O3—C1	−1.2 (5)	Pr2—O4—C2—C1	4.0 (7)
O4W—Pr2—O3—C1	3.6 (6)	O1—C1—C2—O2	−5.0 (8)
O11^iv^—Pr2—O3—C1	−76.4 (5)	O3—C1—C2—O2	175.4 (6)
O10—Pr2—O3—C1	−162.1 (5)	O1—C1—C2—O4	174.6 (5)
O7^iii^—Pr2—O3—C1	−123.3 (5)	O3—C1—C2—O4	−5.0 (9)
O8^iii^—Pr2—O4—C2	−128.4 (4)	Pr1—O5—C3—O8	176.5 (5)
O3—Pr2—O4—C2	−1.8 (4)	Pr1—O5—C3—C4	−2.1 (7)
O9—Pr2—O4—C2	−47.9 (5)	Pr2^vi^—O8—C3—O5	−177.6 (5)
O3W—Pr2—O4—C2	−101.8 (5)	Pr2^vi^—O8—C3—C4	1.0 (7)
O4W—Pr2—O4—C2	−178.4 (5)	Pr1—O6—C4—O7	179.3 (4)
O11^iv^—Pr2—O4—C2	90.4 (4)	Pr1—O6—C4—C3	0.9 (6)
O10—Pr2—O4—C2	28.9 (5)	Pr1^v^—O7—C4—O6	8.5 (9)
O7^iii^—Pr2—O4—C2	137.0 (4)	Pr2^vi^—O7—C4—O6	175.9 (5)
O12^i^—Pr1—O5—C3	85.5 (5)	Pr1^v^—O7—C4—C3	−173.1 (3)
O1W—Pr1—O5—C3	174.2 (5)	Pr2^vi^—O7—C4—C3	−5.7 (6)
O1—Pr1—O5—C3	−146.1 (4)	O5—C3—C4—O6	0.7 (8)
O2—Pr1—O5—C3	125.0 (4)	O8—C3—C4—O6	−178.0 (6)
O2W—Pr1—O5—C3	−88.3 (5)	O5—C3—C4—O7	−177.8 (6)
O7^ii^—Pr1—O5—C3	−46.6 (5)	O8—C3—C4—O7	3.4 (8)
O6—Pr1—O5—C3	1.8 (4)	Pr2—O9—C5—O12	−163.2 (4)
O11^i^—Pr1—O5—C3	41.0 (5)	Pr2—O9—C5—C6	15.3 (7)
O12^i^—Pr1—O6—C4	−76.9 (4)	Pr1^viii^—O12—C5—O9	−168.6 (4)
O5—Pr1—O6—C4	−1.3 (4)	Pr1^viii^—O12—C5—C6	12.9 (7)
O1W—Pr1—O6—C4	−10.7 (5)	Pr2—O10—C6—O11	173.1 (4)
O1—Pr1—O6—C4	120.0 (4)	Pr2—O10—C6—C5	−5.4 (7)
O2—Pr1—O6—C4	−126.7 (4)	Pr2^vii^—O11—C6—O10	9.0 (9)
O2W—Pr1—O6—C4	70.2 (4)	Pr1^viii^—O11—C6—O10	179.4 (5)
O7^ii^—Pr1—O6—C4	146.1 (4)	Pr2^vii^—O11—C6—C5	−172.6 (3)
O11^i^—Pr1—O6—C4	−144.9 (4)	Pr1^viii^—O11—C6—C5	−2.2 (6)
O8^iii^—Pr2—O9—C5	−102.6 (5)	O9—C5—C6—O10	−6.3 (8)
O3—Pr2—O9—C5	77.7 (4)	O12—C5—C6—O10	172.3 (5)
O3W—Pr2—O9—C5	177.1 (5)	O9—C5—C6—O11	175.1 (5)
O4—Pr2—O9—C5	123.9 (4)	O12—C5—C6—O11	−6.4 (8)

Symmetry codes: (i) *x*, *y*+1, *z*; (ii) −*x*+1, *y*−1/2, −*z*+1/2; (iii) *x*−1, *y*−1, *z*; (iv) −*x*, *y*+1/2, −*z*+1/2; (v) −*x*+1, *y*+1/2, −*z*+1/2; (vi) *x*+1, *y*+1, *z*; (vii) −*x*, *y*−1/2, −*z*+1/2; (viii) *x*, *y*−1, *z*.

### Hydrogen-bond geometry (Å, º)

**Table:**

*D*—H···*A*	*D*—H	H···*A*	*D*···*A*	*D*—H···*A*
O1*W*—H1*W*···O2^ix^	0.85	2.02	2.852 (6)	166
O1*W*—H2*W*···O8^x^	0.85	2.15	2.998 (6)	173
O2*W*—H3*W*···O4^xi^	0.85	2.40	2.998 (6)	128
O2*W*—H4*W*···O6^ii^	0.85	2.01	2.792 (6)	152
O3*W*—H5*W*···O12^xii^	0.85	1.97	2.780 (6)	158
O3*W*—H6*W*···O3^xii^	0.85	2.60	3.379 (7)	154
O4*W*—H7*W*···O1^xiii^	0.85	2.16	2.865 (6)	140
O4*W*—H8*W*···O9^xii^	0.85	2.04	2.882 (6)	169

Symmetry codes: (ii) −*x*+1, *y*−1/2, −*z*+1/2; (ix) *x*+1/2, −*y*+3/2, −*z*; (x) *x*−1/2, −*y*+3/2, −*z*; (xi) *x*+1, *y*, *z*; (xii) *x*−1/2, −*y*+1/2, −*z*; (xiii) *x*−1, *y*, *z*.
